# Phytochemicals as modulators of dendritic cell functions: implications for tolerogenic cell-based therapy

**DOI:** 10.3389/fimmu.2025.1653803

**Published:** 2025-11-11

**Authors:** Tünde Fekete, Kitti Pázmándi

**Affiliations:** Department of Immunology, Faculty of Medicine, University of Debrecen, Debrecen, Hungary

**Keywords:** phytochemicals, dendritic cells, bioactive compounds, inflammation, Autoimmune disease, therapy, tolerogenic

## Abstract

Dendritic cells (DCs) constitute a heterogeneous population of immune cells that acting as antigen presenting cells link innate and adaptive immune responses. Their functions are mainly dictated by microenvironmental cues, enabling them to either maintain immune tolerance or initiate robust humoral and cellular immune responses. While DCs are important for orchestrating immune responses, accumulating evidence suggests that aberrant DC activation contributes to the pathogenesis of autoimmune and chronic inflammatory diseases, making them promising targets for therapeutic modulation. Modulating DC functionality therefore represents a potent strategy to attenuate excessive inflammation in such conditions. Plant-derived bioactive compounds, or phytochemicals, are structurally diverse secondary metabolites with established anti-inflammatory and immunomodulatory properties. This review consolidates current *in vitro*, *in vivo*, and *in silico* findings on ten well-characterized phytochemicals including curcumin, 6-gingerol, 6-shogaol, resveratrol, epigallocatechin-3-gallate, quercetin, apigenin, capsaicin, berberine and ginsenosides, which have the capacity to modulate DC phenotype and function. Notably, these phytochemicals can skew DCs toward a tolerogenic phenotype, characterized by reduced expression of antigen presenting and co-stimulatory molecules, diminished pro-inflammatory cytokine secretion, and enhanced regulatory T cell induction. Mechanistic insights reveal convergence on key signaling pathways such as nuclear factor-kappa B (NF-κB), mitogen activated protein kinase (MAPK) and mammalian target of rapamycin (mTOR) in DCs. *In silico* studies further predict interactions of these compounds with various molecular targets, providing a structural basis for their immunoregulatory effects. Furthermore, studies using preclinical models of autoimmune and inflammatory diseases have demonstrated that these phytochemicals can attenuate disease severity, likely through DC modulation. Given their multifaceted immunomodulatory capacity, phytochemicals hold promise both as adjuvant therapies in DC-mediated autoimmune diseases and as agents for generating tolerogenic DCs for cell-based immunotherapies.

## 1 Introduction

Dendritic cells (DCs) are a specialized group of immune cells with a pivotal role in orchestrating both innate and adaptive immune responses. Beyond their well-known function as professional antigen presenting cells, DCs are essential for maintaining peripheral tolerance and immune homeostasis under physiological conditions. As primary sentinels of the immune system, they rapidly detect invading pathogens or endogenous danger signals and initiate appropriate immune responses. Their inherent plasticity enables them to dynamically respond to environmental stimuli and to adopt either immunostimulatory or tolerogenic phenotypes, thus contributing to both initiation of inflammatory responses and the induction of immune tolerance. Owing to their exceptional immunomodulatory capacity, they are considered promising therapeutic targets for the treatment of various inflammatory and autoimmune diseases (1).

In recent years, increasing attention has been directed toward plant-derived bioactive compounds as potential immunomodulatory agents (2). Plants are rich in diverse secondary metabolites, commonly categorized into phenolics, terpenoids, and nitrogen-containing compounds based on their chemical structures, biosynthetic origin, and biological functions (3). These phytochemicals are not only central to the defense mechanisms of the plants, but also exert a wide range of pharmacological effects in humans. Many of them have been recognized for their anti-inflammatory, antioxidant antimicrobial, anticancer, neuroprotective, and cardioprotective activities. Their immunomodulatory properties, in particular, have gained attention in the context of chronic inflammation and immune mediated disorders (4).

Several *in vitro* and preclinical studies have demonstrated that specific phytochemicals can modulate DC function by promoting a tolerogenic phenotype. These compounds are often derived from medicinal and culinary plants such as herbs, spices, fruits, and vegetables, which have long been used in traditional medicine (4). By interfering with signaling pathways, cytokine production, and costimulatory molecule expression, these molecules may inhibit DC maturation or promote the development of DC subsets with a tolerogenic phenotype. Consequently, they hold therapeutic promise in shaping immune responses toward tolerance, especially when applied in *ex vivo* DC-based immunotherapies.

In this review, we aimed to compile current evidence on the immunomodulatory effects of plant-derived bioactive compounds on DCs, with a particular focus on their potential use in treating autoimmune and inflammatory conditions. Specifically, we selected 10 well-characterized phytochemicals including curcumin, 6-gingerol, 6-shogaol, resveratrol, epigallocatechin-3-gallate, quercetin, apigenin, capsaicin, berberine and ginsenosides. These bioactive compounds were selected based on literature data demonstrating their immunosuppressive properties and ability to promote tolerogenic DC differentiation. Scientific publications from the past two decades were collected from databases such as PubMed and Web of Science, using combinations of terms such as “dendritic cell,” “tolerogenic,” “immunosuppressive,” and the specific compound names. Retrieved articles were manually screened to ensure relevance, and reviewed to provide a comprehensive overview of the current findings and to highlight potential directions for future therapeutic applications. In addition, during the manual selection process, preference was given to peer-reviewed articles published in reputable scientific journals to ensure data quality. Studies using purified compounds were included, while those employing crude extracts were excluded. The chemical classification, structural features, and biological functions of the selected compounds are presented in **Table 1**.

**Table 1:** Characteristics and main biological properties of selected phytochemicals.

Compound	Class	Main plant source	2D structure	Biological properties	Ref.
curcumin	polyphenol	*Curcuma longa*	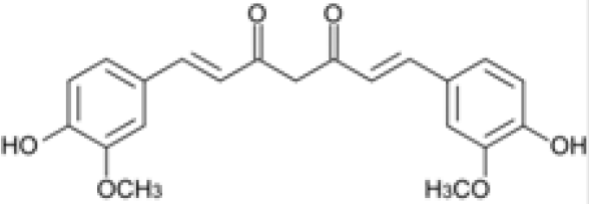	anti-inflammatory antioxidant antimicrobial anticancer	(18)
6-gingerol	phenolic compound	*Zingiber officinale*	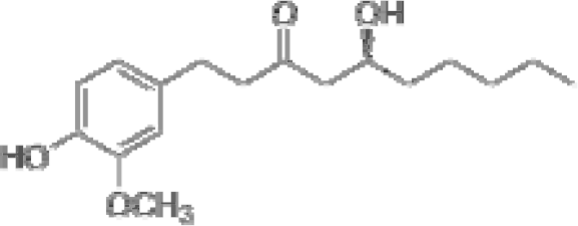	anti-inflammatory antioxidant antimicrobial anticancer antiemetic	(38)
6-shogaol	phenolic compound	*Zingiber officinale*	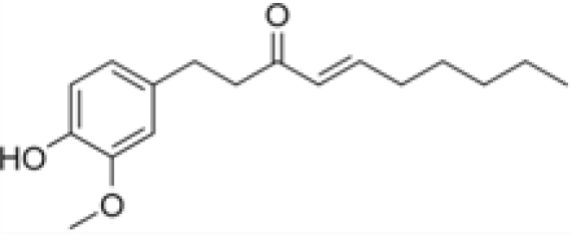	anti-inflammatory antioxidant antimicrobial anticancer antiemetic	(38)
resveratrol	polyphenol	*Polygonum cuspidatum*	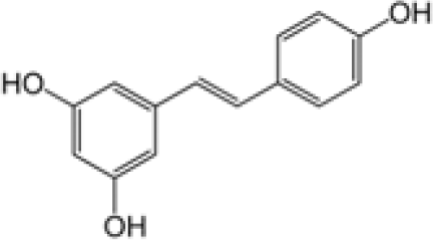	anti-inflammatory antioxidant anticancer	(45)
EGCG	flavonoid	*Camellia sinensis*	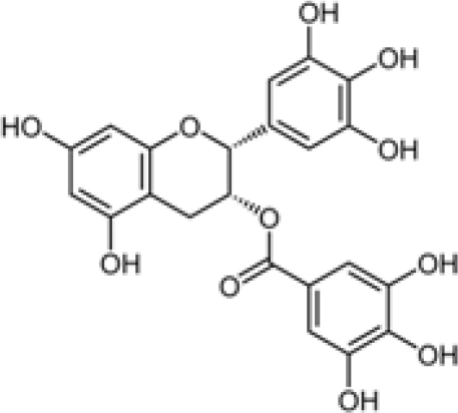	anti-inflammatory antioxidant anticancer	(58)
quercetin	flavonoid	various fruits, vegetables, medicinal plants	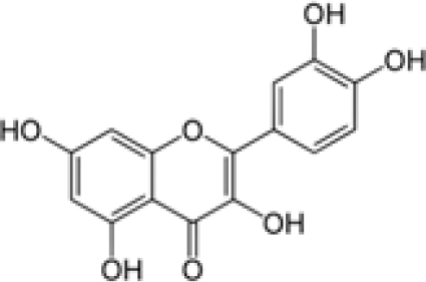	anti-inflammatory antioxidant antimicrobial	(142)
apigenin	flavonoid	*Matricaria recutita*	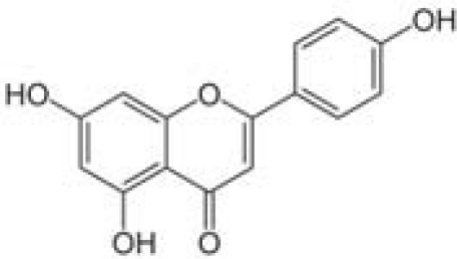	anti-inflammatory antioxidant anticancer anti-diabetic	(80)
capsaicin	alkaloid	*Capsicum annuum*	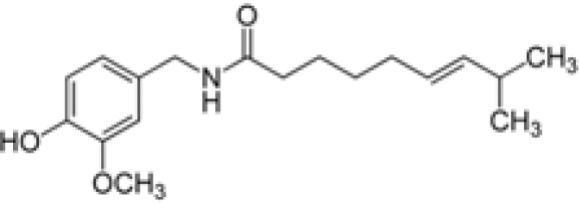	anti-inflammatory antioxidant anticancer analgesic	(87)
berberine	alkaloid	*Berberis* species	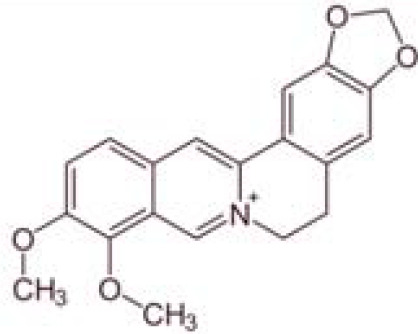	anti-inflammatory antioxidant	(99)
ginsenosides	triterpenoid saponin	*Panax ginseng*	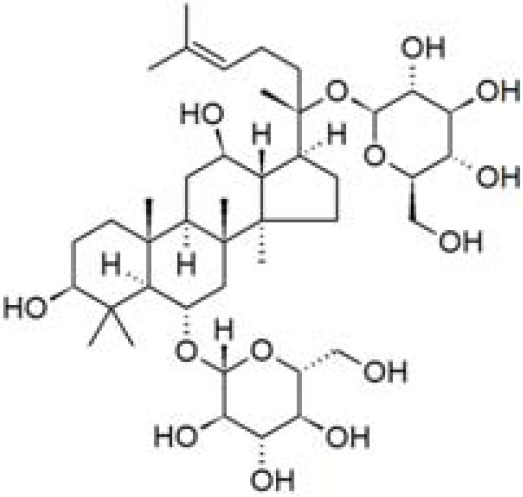	anti-inflammatory antioxidant antimicrobial	(108)

Ref, reference; EGCG, Epigallocatechin-3-gallate.

2D structure of ginsenoside Rg1 is presented.

## 2 DCs at a glance

In the early 1970’s a special stellate shaped cell type was discovered in mouse spleen by Steinmann and Cohn (5, 6). These cells were named DCs for their tree-like processes after the Greek word, dendron, meaning tree. The large surface area enables DCs to interact with numerous surrounding cells, and simultaneously present antigens to multiple T cells. While DCs are known for their high antigen presenting capacity, their role goes far beyond antigen presentation. In addition to interacting with T cells, DCs communicate with innate immune cells such as natural killer (NK) cells and neutrophils, and convey information to non-immune cell types as well. As a result, DCs are able to exhibit a multitude of tasks from initiating, coordinating and regulating immune responses to maintaining tissue homeostasis and self-tolerance (1).

DCs constitute a heterogeneous population of immune cells originating from the bone marrow (7). They are widely distributed throughout the body, circulate in the blood and are present in both lymphoid and non-lymphoid tissues. Based on their ontogeny, phenotype and transcriptional profile DCs can been categorized into distinct subsets. In humans, DCs comprise approximately 1% of peripheral blood mononuclear cells (PBMCs) and are generally classified into two major groups: plasmacytoid DCs (pDCs) and conventional DCs (cDCs), each endowed with distinct effector functions (7). pDCs majorly sense viral infections and as a response rapidly produce type I interferons (IFNs) to create an immediate phase of antiviral state in the surrounding cells (8). The cDC compartment consists of several subtypes that express a broad range of pattern recognition receptors such as Toll-like receptors (TLRs) enabling them to detect a wide variety of pathogens (9). Importantly, under inflammatory conditions, DCs might also arise from monocytes that are usually termed as inflammatory or monocyte-derived DCs (moDCs) (7). In 1994, Sallusto and Lanzavecchia described that human monocytes treated with granulocyte-macrophage colony-stimulating factor (GM-CSF) and interleukin-4 (IL-4) acquire DC-like properties (10). Since then, moDC has become the most commonly used *in vitro* model for studying DC biology and for DC-based immunotherapy approaches. For a long time, Langerhans cells (LCs) in the skin were also considered DCs due to their migratory capacity and antigen presenting functions. However, recent findings have revealed their distinct developmental origin and LCs have been reclassified as macrophages (11). The major DC subsets are highly conserved between mice and humans. Although, several phenotypic differences exist between the same DC subsets across the two species, functional equivalences can often be drawn (12). Therefore, mouse models are the most widely used systems for studying DC biology and evaluating the therapeutic potential of DC-based vaccines (13). The classification, ontogeny and functional specialization of DC subsets have been comprehensively reviewed elsewhere (1, 7).

In general, DCs exist in two functional states: a resting state and an activated state (1). In their resting or quiescent state, DCs act as sentinels of the immune system, continuously surveilling tissues for invading pathogens, damaged or cancerous cells. Resting DCs are characterized by high endocytic capacity, low expression of costimulatory molecules and rapid turnover of major histocompatibility complex II (MHC II) molecules. Importantly, resting DCs constitutively express both MHC I and MHC II molecules, but the dynamics of these molecules on the cell surface differ substantially. MHC II-peptide complexes exhibit rapid turnover on the surface of resting DCs to ensure constant sampling and presentation of extracellular antigens to CD4^+^ T cells (14). In contrast, MHC I-peptide complexes are more stable and long-lived on the plasma membrane that is sufficient for continuous surveillance of intracellular proteins and presentation to CD8^+^ T cells (15). Upon sensing changes in their local environment, DCs become activated, enabling them to migrate to draining lymph nodes and engage with other immune cell types such as B and T cells. Activated DCs are characterized by reduced endocytosis, upregulation of the homing receptor C-C chemokine receptor type 7 (CCR7), upregulation of MHC I, stabilization of MHC II expression, and increased capacity to prime naive T cells. According to the latest concept, DC activation is a prerequisite for communication with other cells, and depending on the stimuli encountered, they can initiate either tolerogenic or immunogenic immune responses (1). Immunogenic DCs promote the differentiation of naive T cells into effector subsets, such as T helper (Th) 1 and Th17 cells, thereby they are highly efficient in facilitating adaptive immunity against invading pathogens and tumors. In contrast, tolerogenic DCs support the development of regulatory T cells (Tregs), which exert suppressive functions, and are essential for maintaining immune tolerance and prevention of autoimmunity (16).

Nevertheless, altered distribution and aberrant activation of DCs might lead to the breakdown of immune tolerance against self-antigens and result in the induction of autoimmune disorders such as systemic lupus erythematosus (SLE), rheumatoid arthritis (RA), and psoriasis (17). Consequently, modulation of DC functionality has emerged as a promising therapeutic approach to treat autoimmune conditions.

## 3 Effects of plant-derived compounds on DCs

Natural bioactive compounds are commonly found in healthy foods such as fruits and vegetables, as well as in beverages like herbal and green teas, which are consumed daily by millions of people (4). This chapter focuses on ten well-studied plant-derived compounds that have gained attention due to their anti-inflammatory, antimicrobial and antioxidant properties. Numerous studies have investigated the impact of these phytochemicals on various compartments of the immune system. Here, we aimed to highlight the effects of these selected natural compounds on DCs and introduce the general mechanisms underlying their activities. Their immunomodulatory effects, mechanisms of action on DCs, and predicted targets based on *in silico* studies are summarized in **Table 2**.

**Table 2:** The immunomodulatory effects, the mechanisms of action on DCs, and predicted molecular targets of selected phytochemicals.

Compound	Observed effects in DCs	Mechanism of action in DCs (*in vitro*)	Target (in silico)	Ref. (in silico)
curcumin	MHC II and costimulatory molecules↓ inflammatory cytokines and chemokines↓ migration↓ endocytic capacity↑ T cell proliferation and Th1/Th17 polarization ability ↓ Treg priming ability↑	NF-κB, MAPK, and mTOR inhibition NRF2 activation AMPK activation mGluR4 upregulation	EGFR, NF-κB DHFR MMP3 NLRP3 COX-2 MD-2 TLR4, IRAK1, caspase-3	(31) (32) (33) (34) (35) (36) (37)
6-gingerol	MHC II and costimulatory molecules↓ inflammatory cytokines↓ Th1/Th17 polarization ability↓	NF-κB, MAPK, and mTOR inhibition	5-LOX COX1	(41) (43)
6-shogaol	MHC II and costimulatory molecules↓ inflammatory cytokines↓ Th1 polarization ability↓	NF-κB, MAPK, and mTOR inhibition NRF2 activation AMPK activation	5-LOX KEAP1, GSK-3β	(41) (42)
resveratrol	MHC I, MHCII, and costimulatory molecules↓ inflammatory cytokines, ROS↓ T cell proliferation ability↓ IL-10↑	NF-κB and MAPK inhibition ILT3, ILT4 upregulation	TLR4, IRAK1, caspase-3 MMP2, MMP9	(37) (57)
EGCG	MHC II and costimulatory molecules↓ inflammatory cytokines↓ T cell proliferation ability↓ COX-2, IDO, PGE_2_↓ endocytic capacity↑ IL-10↑	NF-κB and MAPK inhibition STAT1 inhibition Tollip upregulation through 67LR	TLR4, IRAK1, caspase-3 NF-κB IKKβ	(37) (66) (67)
quercetin	MHC II, CCR7, and costimulatory molecules↓ inflammatory cytokines and chemokines↓ migration↓ endocytosic capacity↓ T cell proliferation and Th1/Th17 polarization ability↓ Treg priming ability↑	NF-κB, MAPK and Akt inhibition Ahr activation Dab2, ILT3, ILT4, ILT5, CD39, CD73 upregulation STAT4 inhibition	TLR4, IRAK1, caspase-3 IKKβ, SOD DAPK1	(37) (77) (78)
apigenin	MHC I, MHC II, CCR7, and costimulatory molecules↓ inflammatory cytokines, IFN-α↓ IL-10 and TGF-β↑ migration↓, COX-2↓ endocytic capacity↑ T cell proliferation and Th1/Th17 polarization ability↓ Treg priming ability↑	NF-κB and MAPK inhibition	IKK, NF-κB, p38, COX-2	(86)
capsaicin	mouse DC: MHC II, costimulatory molecules and inflammatory cytokines ↑ human DC: CD83, CCR7 and inflammatory cytokines ↓	TRPV1 activation	TRPV1 HSP90 c-Abl, p38, c-Src kinase, VEGFR COX-2, IL-6, TGF-β	(94) (95) (96) (97)
berberine	costimulatory molecules↓ inflammatory cytokines↓ IDO, TGF-β↑ T cell proliferation and Th1/Th17 polarization ability↓ Treg priming ability↑	not investigated	KKα AMPK	(106) (107)
ginsenoside	MHC II, CCR7 and costimulatory molecules↓ inflammatory cytokines↓ T cell proliferation and Th1 polarization ability↓ efferocytosis↓ migration↓ Treg priming ability↑	not investigated	SLC7A11 TLR4 PPARγ Annexin A2	(115) (118) (119) (120)

5-LOX, 5-lipoxygenase; 67LR, 67 kDa laminin receptor; AMPK, AMP-activated protein kinase; CCR7, C-C chemokine receptor type 7; CD, Cluster of differentiation; COX, cyclooxygenase; DAPK1, death-associated protein kinase 1; DC, dendritic cell; DHFR, dihydrofolate reductase; EGFR, epidermal growth factor receptor; GSK-3β, glycogen synthase kinase 3-β; HSP90, heat-shock protein 90; IDO, indoleamine 2,3-dioxygenase; IFN, interferon; IKK, IκB kinase; IL, interleukin; ILT, immunoglobulin-like transcript; IRAK1, interleukin-1 receptor–associated kinase 1; KEAP1, Kelch-like ECH-associated protein 1; MAPK, mitogen-activated protein kinase; MD-2, myeloid differentiation protein 2; mGluR4, metabotropic glutamate receptor-4; MHC, major histocompatibility complex; MMP, matrix metalloproteinase; mTOR, mechanistic target of rapamycin, NF-κB, nuclear factor-kappa B; NLRP3, NOD-, LRR-, and pyrin domain-containing protein 3; NRF2, nuclear factor erythroid 2-related factor 2; PGE_2_, prostaglandin E2; PPARγ, peroxisome proliferator-activated receptor gamma; Ref, reference; ROS, reactive oxygen species; SLC7A11, Solute carrier family 7 member 11; SOD, superoxide dismutase; TGF-β, transforming growth factor-beta; Th, T helper cell; TLR4, Toll-like receptor 4; Tollip, Toll interacting protein; TRPV1, transient receptor potential vanilloid type 1; Treg, regulatory T cell; VEGFR, vascular endothelial growth factor receptor.

### 3.1 Curcumin

Turmeric (*Curcuma longa*), a member of the *Zingiberaceae* family, has been used as both a culinary spice and traditional herbal medicine for thousands of years in India and Eastern Asia (18). Curcumin, the main bioactive compound in its rhizome, has been extensively studied for its diverse pharmacological properties over the last few decades. Several *in vitro* and *in vivo* animal studies, as well as clinical trials, have demonstrated its anticancer, anti-inflammatory, and radioprotective properties, as summarized in a recent review (19).

Curcumin is one of the most thoroughly investigated phytochemicals, with its effect on DCs first reported in 2005 (20). In murine bone marrow-derived DCs (BM-DCs) stimulated with the TLR4 ligand lipopolysaccharide (LPS), curcumin significantly decreased the expression of MHC II and the costimulatory molecules CD80 and CD86 (20). Furthermore, it suppressed the LPS-triggered production of various inflammatory cytokines such as interleukin (IL)-12, IL-1β, IL-6 and tumor necrosis factor (TNF), and reduced the capacity of DCs to elicit a Th1 response. Interestingly, curcumin-treated DCs exhibited increased endocytic capacity, suggesting that curcumin maintains DCs in a resting state. These effects were associated with curcumin-mediated inhibition of central signaling pathways such as the nuclear factor-kappa B (NF-κB) and mitogen activated protein kinase (MAPK) cascades in DCs. In particular, pre-treatment with curcumin prevented the LPS-triggered nuclear translocation of NF-κB and the phosphorylation of the p38, c-Jun N-terminal Kinase (JNK) and extracellular signal-regulated kinase (ERK) (20). Similar results were observed in human moDCs, where pre-treatment with curcumin inhibited the LPS and polyinosinic:polycytidylic acid (polyI:C)-induced upregulation of different activation markers (CD83, CD86, and HLA-DR) and the secretion of various cytokines including IL-12, TNF and IL-10 (21). Furthermore, curcumin reduced endocytosis and the ability of DCs to prime T cell proliferation. Additionally, curcumin significantly reduced the production of the chemokine CXCL10 and prevented the migration of DCs in response to CCL19 and CCL21, further suggesting its immunosuppressive potential (21). Further studies confirmed that curcumin promotes the development of tolerogenic DCs. Curcumin-treated murine BM-DCs generated from B6 mice show increased IL-10 production, and support the differentiation of Tregs from naive CD4^+^ T cells (22). Additionally, transforming growth factor-β (TGF-β) and retinoic acid produced by curcumin-treated DCs were also required to induce Treg differentiation.

Mechanistically, curcumin induces an anti-inflammatory phenotype in DCs by activating nuclear factor-erythroid 2-related factor 2 (NRF2) and promoting the expression of heme oxygenase 1 (HO-1) (23, 24). Brück et al. showed that curcumin induced HO-1 expression and signal transducer and activator of transcription 3 (STAT3) phosphorylation in LPS-activated mouse BM-DCs thereby inhibiting their ability to promote Th1 and Th17 polarization through repression of *IL-12b* and *IL-23a* transcription. Similarly, curcumin was also able to inhibit the functionality of LPS-stimulated human moDCs through upregulating the immunomodulatory enzyme, HO-1 (24). In a subsequent study the authors also demonstrated that upregulation of HO-1 is dependent on the activation of AMP-activated protein kinase (AMPK). Generally, AMPK negatively regulates the mammalian target of rapamycin (mTOR) signaling cascade, thus inhibiting the glycolytic reprogramming required for full DC activation (25). Furthermore, curcumin inhibited Th17 differentiation by suppressing the production of IL-6 and IL-23 in mouse BM-DCs (26). This effect might be associated with the upregulation of metabotropic glutamate receptor-4 (mGluR4), a molecule known to favor Treg development, and thus inhibit autoimmunity (26).

It was also demonstrated that curcumin-treated human moDCs and murine CD11c+ DCs promote Treg differentiation and expansion both *in vitro* and *in vivo* (27). These findings were corroborated by *in vivo* experiments demonstrating that Tregs generated in the presence of curcumin-treated DCs mitigated Th1-mediated colitis in mice (22). In a mouse model of inflammatory bowel disease (IBD), curcumin alleviated disease symptoms as evidenced by reduced colonic damage and decreased inflammatory cell infiltration to the colonic mucosa (28). Moreover, curcumin not only decreased the number of CD11c^+^/MHC II^+^ DCs in the Peyer patches, but also reduced the level of different costimulatory molecules on their surface (28). Additionally, tetramethylcurcumin, an analog of curcumin, was shown to stimulate the release of immunosuppressive extracellular vesicles (EVs) from BM-DCs cultured with ovalbumin (29). Intranasal administration of these EVs induced Treg differentiation in a mouse model of allergic rhinitis thereby alleviating disease symptoms and inflammation (29). In patients with psoriasis, curcumin reduced the expression of IL-17, GM-CSF and IFN-γ in PBMCs further supporting its therapeutic relevance (30).

*In silico* studies have elucidated potential molecular targets that may underlie the anti-inflammatory and antioxidant effects of curcumin. Strong binding to both epidermal growth factor receptor (EGFR) and NF-κB suggests a synergistic blockade of upstream receptor-mediated and downstream transcriptional inflammatory signaling (31). Inhibition of EGFR signaling can attenuate pro-inflammatory cellular responses by reducing activation of MAPK and STAT pathways, while suppression of NF-κB activity decreases transcription of inflammatory cytokines. Molecular docking results indicate that curcumin binds to dihydrofolate reductase (DHFR) with an affinity comparable to methotrexate, a clinically established immunosuppressive agent for RA (32). Curcumin also shows binding potential to matrix metalloproteinase 3 (MMP-3), a protease whose expression and activity are elevated in chronic inflammatory diseases, including RA (33). MMP3 inhibition may prevent extracellular matrix degradation and inflammatory tissue damage. Docking studies have revealed that curcumin can inhibit the NOD-, LRR- and pyrin domain-containing protein 3 (NLRP3) inflammasome, potentially reducing caspase-1 activation and subsequent IL-1β maturation (34). *In silico* analyses of 15 curcumin analogues show binding to multiple subdomains of cyclooxygenase-2 (COX-2), a pivotal enzyme in prostaglandin synthesis during inflammation (35). Molecular docking and dynamics simulations suggest that curcumin can embed into the hydrophobic pocket of myeloid differentiation protein 2 (MD-2), a co-receptor for TLR4 that mediates lipopolysaccharide (LPS)-induced signaling, thereby dampening TLR4-mediated activation (36). Further, *in silico* investigations suggest that curcumin along with quercetin, EGCG, and, resveratrol can inhibit TLR4-mediated signaling through directly interacting with TLR4 and IL-1 receptor-associated kinase 1 (IRAK1), a kinase downstream of TLR-4 (37). These compounds were further predicted to interact with caspase-3, which is primarily known for its role in apoptosis but also plays a significant role in regulating inflammation (37).

### 3.2 Gingerols and shogaols

Ginger (*Zingiber officinale*), like turmeric, belongs to the *Zingiberaceae* family and has a long history of use as both a spice and medicinal plant. Its rhizome is rich in biologically active compounds, such as gingerols and shogaols, which are primarily responsible for its therapeutic effects. 6-gingerol is the principal active compound in fresh ginger, whereas 6-shogaol predominates in dried preparations (38). While numerous studies have shown that gingerols and shogaols influence the function of various immune cell types, only two reports have explored their effects on DCs.

Our group has recently demonstrated that both 6-gingerol and 6-shogaol modulate the functionality of human moDCs (39). Both phenolic compounds decreased the expression of CD40, CD83, CD86 and HLA-DQ, as well as reduced the secretion of TNF, IL-6 and IL-10 cytokines by TLR-stimulated moDCs. Furthermore, both compounds could significantly reduce the inflammatory cytokine production in moDCs triggered by *Escherichia coli*, and thus their capacity to promote Th1 differentiation from naive CD4^+^ T cells. Our mechanistic studies revealed that 6-gingerol and 6-shogaol interfered with the TLR-mediated activation of NF-κB, MAPK and mTOR signaling cascades in moDCs. Further, we demonstrated that 6-shogaol, but not 6-gingerol, enhanced AMPK phosphorylation, and activated the NRF2/HO-1 axis suggesting its superior anti-inflammatory potential compared to 6-gingerol (39).

Another study demonstrated that 6-gingerol induced a tolerogenic phenotype in LPS-stimulated mouse BM-DCs as indicated by low expression of MHC II and costimulatory molecules, reduced cytokine secretion, and impaired ability to prime Th17 cells. In agreement with our results, it was also demonstrated that 6-gingerol suppressed DC functionality by modulating the NF-κB and MAPK signaling pathways in LPS-stimulated mouse BM-DCs. *In vivo*, 6-gingerol significantly ameliorated the severity of experimental autoimmune encephalomyelitis (EAE), a murine model of multiple sclerosis (MS), by inhibiting DC activation and Th17 polarization. Furthermore, 6-gingerol significantly inhibited inflammatory cell infiltration and demyelination in the central nervous system, and lowered the frequencies of CD11c^+^CD80^+^ activated DCs and Th17 cells in the spleen (40).

*In silico* studies have identified several molecular targets that may contribute to the anti-inflammatory and antioxidant properties of 6-gingerol and 6-shogaol. Both compounds can interact with 5-lipoxygenase (5-LOX) (41), an enzyme responsible for leukotriene biosynthesis, suggesting a role in suppressing lipid mediator–driven inflammation. Thiophene derivatives of 6-shogaol have been predicted to activate the NRF2 antioxidant pathway by binding to key regulators, including Kelch-like ECH-associated protein 1 (KEAP1) and glycogen synthase kinase 3-β (GSK-3β) that might lead to the upregulation of cytoprotective and anti-inflammatory genes (42). Additionally, 6-gingerol derivatives showed binding ability to cyclooxygenase-1 (COX-1) (43), indicating potential to reduce prostaglandin-mediated inflammatory responses.

### 3.3 Resveratrol

Resveratrol is a polyphenol that has various sources such as berries, grapes, red wine and peanuts. When sold as a food supplement, resveratrol is predominantly extracted from Japanese knotweed (*Polygonum cuspidatum*), a plant traditionally used in East Asian medicine (44). Accumulating evidence suggests its anti-inflammatory and immunomodulatory effects, including its protective role in respiratory system diseases such as asthma and chronic obstructive pulmonary disease (COPD), partly through its action on DCs (45).

In 2004, Kim et al. demonstrated that resveratrol inhibited LPS-mediated activation of BM-DCs *in vitro*. Resveratrol significantly reduced the production of IL-12 and the surface expression of CD80, CD86, MHC I and MHC II molecules, while preserving the endocytic capacity of DCs. Consequently, these DCs also exhibited decreased T cell stimulatory capacity (46). Similar results were obtained when resveratrol was added to human moDCs (47, 48). Specifically, resveratrol decreased the nuclear translocation of NF-κB p65, the production of IL-12 as well as the expression of CD40, CD80 and CD86 costimulatory molecules in LPS-stimulated human moDCs (47). In contrast, resveratrol markedly upregulated immunoglobulin-like transcript (ILT) 3 and ILT4, which deliver inhibitory signals and promote a tolerogenic phenotype in DCs (47). Therefore, resveratrol-treated moDCs were poor stimulators of CD4^+^ T cell proliferation and migration. Interestingly, resveratrol-treated moDCs promoted the proliferation of IL-10 secreting T cells but failed to induce forkhead box P3 (FOXP3) expression. Comparable findings were reported in TNF-stimulated moDCs (48), where resveratrol reduced NF-κB p65 nuclear translocation, CD83 and CD86 expression, and IL-12 and IL-23 production, while increasing IL-10 levels. Furthermore, resveratrol also suppressed the capacity of TNF-stimulated DCs to initiate CD3^+^ T cell proliferation. Resveratrol also blocked moDC activation in response to advanced glycation end products (AGEs) (49), known to contribute to the pathogenesis of autoimmune diseases via DC activation (50). In particular, resveratrol inhibited the expression of various activation markers, costimulatory molecules, and the receptor for AGE (RAGE) and suppressed the production of inflammatory cytokines in moDCs stimulated with glycated albumin. It also reduced the activation of the MAPK and NF-κB pathways, and impaired the allostimulatory potential of moDCs (49).

A recent study demonstrated that the immunomodulatory effect of resveratrol depends highly on its structural features and mode of delivery. Hydroxylated and methylated derivatives are characterized by different biological activities compared to the parent compound (51). Resveratrol and its hydroxylated derivative, piceatannol, potently inhibited the LPS-induced production of reactive oxygen species (ROS), whereas the methylated monomers of resveratrol showed a reduced antioxidant capacity in mouse BM-DCs (51). These compounds also differ in their potential to elicit cytokine production by BM-DCs in response to LPS and bacteria implying the importance of structure with regards to the immunomodulatory potential of resveratrol. Another study demonstrated that resveratrol encapsulated in nanostructured lipid carriers (NLC) more effectively suppressed TNF-induced CD83 expression, IL-12 and IL-23 production, and NF-κB p65 phosphorylation than free resveratrol in moDCs (52).

Subsequent studies further demonstrated that resveratrol can alleviate lung inflammation by modulating DC functions. *In vivo*, resveratrol ameliorated acute lung injury (ALI) in mice challenged intratracheally with LPS (53). Resveratrol pre-treatment greatly reduced the levels of pro-inflammatory cytokines, including, TNF, IL-6, IL-12, whereas increased the levels of anti-inflammatory cytokines, including TGF-β, IL-10, IL-13 and IL-33 in the bronchoalveolar lavage fluid (BALF) of ALI mice. Resveratrol dramatically decreased the expression of CD80, CD86 and MHC II, the production of IL-12, while increased ILT3 expression and IL-10 secretion in pulmonary and splenic cDCs of ALI mice. *In vitro*, resveratrol pretreatment of BM-DCs inhibited LPS-induced activation, cytokine production, and T cell stimulatory capacity of DCs as well (53).

Human moDCs differentiated from monocytes of chronic obstructive pulmonary disease (COPD) patients expressed increased levels of CD80, CD86 and IFN-α as compared with those of healthy individuals (54). Resveratrol pre-treatment reduced the level of these molecules, possibly via downregulation microRNA-34, an important regulator of inflammatory responses (55).

A subsequent study investigated how the stability, bioavailability and thus the bioactivity of resveratrol could be enhanced by irradiation. Intriguingly, γ-irradiated resveratrol has a lower toxicity compared to its intact form, and has strong immunosuppressive properties as it can significantly inhibit inflammatory cytokine production, costimulatory molecule expression, and antigen-presentation by LPS-activated mouse BM-DCs (56). It also promoted IL-10 production and Treg production when added to differentiating BM-DCs.

Oral administration of γ-irradiated resveratrol attenuated the clinical signs of colitis in DSS-treated mice, suggesting the therapeutic potential of γ-irradiated resveratrol in IBD (56). However, the lack of direct functional comparison of γ-irradiated resveratrol to its unmodified form limits conclusions regarding its superior ability to induce tolerogenic DCs.

*In silico* studies have revealed that TLR4, IRAK1, and caspase-3 are molecular targets of resveratrol (37). In addition, MMP-2 and MMP-9 have been reported as targets, whose inhibition may reduce extracellular matrix degradation and attenuate inflammation (57).

### 3.4 Epigallocatechin-3-gallate

Green tea (*Camellia sinensis*) is a popular beverage that is consumed worldwide for its health benefits. EGCG is a flavonoid that represents the major bioactive compound in green tea and is known for its strong antioxidant capacity (58). Moreover, it has gained a great attention for its anti-inflammatory and immunomodulatory potential.

The immunomodulatory effect of EGCG has been first investigated on mouse BM-DCs (59). This study showed that EGCG effectively inhibits DC functions, as it was able to inhibit IL-12 production and downregulate the expression of CD80, CD86, MHC I and MHC II molecules in LPS-stimulated murine BM-DCs. EGCG-treated DCs were poor inducers of T cell proliferation and activation. The authors suggested that EGCG antagonized the LPS-mediated functionality of DCs by suppressing MAPK and NF-κB activation. Another study demonstrated that EGCG can inhibit IL-12 production while increasing TNF secretion in BM-derived DCs stimulated with LPS, muramyl-dipeptide or *Legionella pneumophila* (60). However, the divergent effects of EGCG on IL-12 and TNF production have not been elucidated yet. In the same year, it was also shown that EGCG pretreatment suppressed COX-2 expression, prostaglandin E2 (PGE_2_) and indoleamine 2,3-dioxygenase (IDO) production in BM-DCs in response to IFN-γ, likely through STAT1 inhibition (61). In human moDCs, EGCG exhibited similar anti-inflammatory effects by attenuating TLR4-mediated signaling. In LPS-stimulated moDCs, EGCG reduced the expression of HLA-DR, CD80 and CD83, and impaired their ability to promote T cell proliferation (62). Conversely, EGCG increased IL-10 production and the endocytic ability of LPS-stimulated DCs suggesting that EGCG maintains DCs in their resting state (62). Experiments performed with murine BM-DCs further demonstrated that the 67 kDa laminin receptor (67LR) is essential for mediating the anti-inflammatory actions of EGCG in DCs (63). EGCG reduced the LPS-induced production of IL-1β, IL-6 and TNF, the expression of CD80, CD86, MHC I and MHC II molecules, and NF-κB and MAPK activation in murine BM-DCs. Interestingly, the inhibitory effect of EGCG was abrogated upon pre-treatment with anti-67LR antibodies, suggesting that the inhibitory actions of EGCG are mediated through 67LR. In addition, EGCG elevated the expression of Toll-interacting protein (Tollip), a negative regulator of TLR signaling through 67LR suggesting that the anti-inflammatory actions of EGCG might be partially mediated by Tollip upregulation in DCs.

Previously it was reported that EGCG”Me, a 3-O-methylated derivative of EGCG, significantly reduced TLR4 expression and thereby exerted anti-inflammatory effects in mouse peritoneal macrophages (64). On the contrary, EGCG3”Me supplementation increased TLR5 expression on lamina propria DCs and macrophages, and enhanced vaccine-induced immune response in mice immunized with a split influenza vaccine (65). Although, EGCG treatment showed overall beneficial effects, the contradiction between its immunosuppressive and immunostimulatory effects warrants further investigation.

Molecular docking and modeling revealed that EGCG can directly bind to TLR4, IRAK1 and caspase-3 (37). *In silico* studies further predicted NF-κB as a principal target of EGCG, suggesting that its anti-inflammatory effects may be mediated through suppression of NF-κB activation and downstream cytokine production (66). Furthermore, EGCG was found to interact with IκB kinase β (IKKβ), a key regulator of NF-κB signaling, indicating that EGCG may inhibit the phosphorylation and degradation of IκB, thereby preventing NF-κB nuclear translocation and inflammatory gene transcription (67).

### 3.5 Quercetin

Quercetin is a flavonoid found in various fruits, vegetables, and medicinal plants. It exhibits a broad range of bioactivities, including antioxidant and anti-inflammatory effects, and has been suggested to alleviate allergy symptoms (68). Several studies indicate that quercetin decreases inflammation primarily by inhibiting DC activation. It also acts as a natural, albeit indirect, ligand for the aryl hydrocarbon receptor (Ahr), which plays a key role in regulating immune responses and promoting tolerogenic properties in DCs (69).

*In vitro* studies demonstrated that quercetin significantly reduced the expression of MHC II and different costimulatory molecules (CD40, CD80, CD86) as well as the production of various cytokines (IL-1 α/β, IL-6, IL-10, IL-12) and chemokines (MCP-1, MIP-1α/β, RANTES) in LPS-stimulated mouse BM-DCs (70). Quercetin also blocked endocytosis by resting DCs and suppressed LPS-stimulated migration of DCs both *in vitro* and *in vivo*. Furthermore, quercetin abrogated T cell activation and proliferation induced by LPS-stimulated BM-DCs. The study also demonstrated that quercetin blocked the LPS-triggered activation of ERK, JNK, Akt and the degradation of IκB indicating that quercetin suppresses DC activation via interfering with the MAPK, Akt and NF-κB signaling pathways (70).

In human moDCs, quercetin impaired the LPS-mediated production of IL-12 and the upregulation of CD83, CD86, HLA-DR and CCR7 (71). It was revealed that quercetin downregulated CD83 through facilitating direct binding of Ahr to the CD83 promoter region. In addition, quercetin decreased the ability of DCs to activate T cells, whereas increased their potential to initiate Treg differentiation in coculture with naive T cells. The data showed that quercetin induced a tolerogenic phenotype in DCs via upregulating various immunomodulatory molecules, including Disabled-2 (Dab2), ILT3, ILT4 and ILT5 inhibitory receptors, and the ATP-degrading ectoenzymes CD39 and CD73 (71). Blockade of Dab2, a negative regulator of intracellular signaling reversed the inhibitory effects of quercetin on BMDC activation suggesting that the regulatory effects of quercetin might be mediated via Dab2 upregulation (72).

Co-administration with piperine, a known bioenhancer, was shown to augment the anti-inflammatory potential of quercetin (73). Genome-wide transcriptome analysis revealed that quercetin and piperine delivered via reconstituted oil bodies (ROBs-QP) significantly downregulated various inflammatory mediators, and decreased the expression of molecules associated with antigen presentation and activation in mouse BM-DCs (74). DCs exposed to ROBs-QP failed to upregulated CCR7, migrate to lymph nodes and efficiently present antigens to naive T cells (74). Intraperitoneal delivery of ROBs-QP also ameliorated DSS-induced colitis symptoms in mice highlighting its potential for treating inflammatory diseases (73). Another report showed that quercetin administration reduced atherosclerosis progression in apolipoprotein E knock out mice by suppressing DC activation (72). Immunohistochemical analysis revealed that quercetin reduced DC and macrophage accumulation in atherosclerotic lesions, and decreased serum IL-6 and IL-12, while increased IL-10 levels. *In vivo*, quercetin also alleviated contact hypersensitivity response elicited via injection of 2,4-dinitrofluorobenzene (DNFB)-pulsed DCs to mice, indicating that quercetin could be used to prevent delayed-type hypersensitivity (70). Furthermore, a recent study demonstrated that quercetin is also able to suppress neuroinflammation in EAE mice by inhibiting DC activation and Th1/Th17 cell differentiation (75). The experimental results suggest that this effect was mediated through inhibition of STAT4 (75), a transcription factor central to DC activation and implicated in autoimmune disease pathogenesis (76).

These findings demonstrate that quercetin efficiently inhibits LPS-induced DC activation, and attenuates different types of inflammatory reactions *in vivo*, suggesting its potential as a therapeutic agent to treat various DC-mediated inflammatory conditions.

Molecular docking analysis revealed that quercetin has considerable binding affinity to IKKβ, a core component of the NF-κB signaling pathway, as well as to the antioxidant enzyme superoxide dismutase (SOD) (77), suggesting that its anti-inflammatory effects are mediated largely through suppression of NF-κB signaling and modulation of oxidative stress. Furthermore, *in silico* studies have indicated Death-Associated Protein Kinase 1 (DAPK1) as a potential molecular target for quercetin and its analogs, implicating possible neuroprotective and anti-inflammatory roles through modulation of DAPK1 activity (78).

### 3.6 Apigenin

Apigenin is a ubiquitous flavonoid synthetized by many different types of plants. One of the richest natural sources of apigenin is chamomile (*Matricaria recutita*), which has been traditionally consumed as an herbal tea to reduce anxiety and treat gastrointestinal complaints (79). Like many flavonoids, apigenin has also been reported to reduce inflammation and oxidative stress (80). Apigenin also has an impact on the immune system by modulating the biological activities of various immune cells, including DCs.

In mouse BM-DCs, apigenin significantly suppressed the LPS-induced production of IL-12 and the expression of CD80, CD86, MHC I, and MHC II molecules (81). In addition, apigenin-treated DCs showed reduced ability to initiate allogeneic T cell proliferation and Th1 differentiation, while displayed high endocytic capacity suggesting that apigenin maintained DCs in their resting state. Apigenin also blocked the NF-κB and MAPK signaling pathways in mouse BM-DCs (81). It also suppressed TLR7- and TLR9-mediated IL-6 and IFN-α production by spleen DCs of lupus mice (82). In human blood derived DCs, apigenin reduced the LPS-triggered expression of CD40, CD83, CD86, CCR7, and MHC I and MHC II molecules, as well as production of IL-1β, IL-6, IL-12 and IL-23 while inducing secretion of IL-10 and TGF-β (83). Consequently, apigenin decreased the ability of LPS-triggered DCs to induce Th1 and Th17 polarization while increased the number of Tregs. In LPS-stimulated DCs, apigenin prevented the nuclear translocation of RelB, a transcription factor, which is involved in the non-canonical activation of the NF-κB pathway and thus in the control of DC activation (83).

*In vivo*, apigenin suppressed splenic DC activation and IFN-γ production of splenic CD4^+^ T cells in LPS-challenged mice and decreased the ability of trinitrobenzene sulfonic acid (TNBS)-pulsed murine BM-DCs to induce contact hypersensitivity (81). In SNF1 mice, a spontaneous lupus mouse model, apigenin reduced COX-2 expression in immune cells including DCs (82). Apigenin also reduced nucleosome-induced IFN-γ and IL-17 response by splenic T cells and autoantibody production of splenic B cells of SNF1 mice. Intraperitoneal injection of apigenin also decreased serum autoantibody levels and delayed the development of glomerulonephritis. In collagen induced arthritis (CIA) mice, an animal model of RA, apigenin reduced joint inflammation, swelling and destruction, most likely via inhibition of DC functions (84). In CIA mice, apigenin reduced levels of TNF, IL-1β and IL-6 in the serum and supernatants from the lymph nodes, and blocked DC activation as shown by reduced expression of costimulatory molecules and MHC II in CD11c^+^ cells. Apigenin also reduced DC migration to the draining lymph nodes of CIA mice (84). These data indicate that apigenin exerts its immunosuppressive effect in arthritis by inhibiting DC activation and migration. Apigenin also reduced the severity of EAE *in vivo* both in progressive (C57BL/6) and relapse-remitting (SJL/J) mouse models of MS. Apigenin decreased immune cell infiltration and reduced demyelination in the CNS of EAE mice while retaining immune cells in the periphery including the blood, spleen and lymph nodes. Apigenin increased the number of CD11c^+^ DCs in all 3 peripheral compartments, and downmodulated the levels of MHC II and CD86 on splenocyte-derived DCs (83). It also reduced the surface expression of α4 integrin on the surface of splenic DCs and CD4^+^ T cell from EAE mice, thereby decreasing their ability to cross the blood-brain barrier (85).

A recent *in silico* study have further identified the molecular targets of apigenin involved in inflammatory pathways, including IKK, the NF-κB p50–p65 heterodimer, p38 MAPK, and COX-2 (86). By modulating these key signaling molecules, apigenin might exert broad anti-inflammatory effects through inhibition of NF-κB activation, MAPK signaling, and prostaglandin synthesis, thereby contributing to the regulation of DC functions.

### 3.7 Capsaicin

Chili pepper (*Capsicum annuum*) is a commonly used spice that has also various health-promoting attributes such as anti-inflammatory, antioxidant, and antimicrobial properties (87). Capsaicin is a naturally occurring alkaloid in chili pepper responsible for its pungent taste, as well as many of its health benefits. Although, several studies have reported beneficial effects of capsaicin in various autoimmune diseases (reviewed in (88)), its effects on DCs remained controversial and its immunomodulatory mechanisms are not completely elucidated.

Initial studies demonstrated that mouse BM-DCs express the capsaicin receptor transient receptor potential channel vanilloid type 1 (TRPV1), and engagement of this receptor by capsaicin promotes DC activation (89). Capsaicin increased surface expression of MHC II and CD86 that could be inhibited by the TRPV1 antagonist capsazepine. Intradermal injection of capsaicin led to the migration of DCs to the draining lymph nodes in TRPV1 expressing mice, but not in TRPV1 deficient animals (89). Studies in human DCs also confirmed TRPV1 expression; however, unlike in mouse BM-DCs, capsaicin exerted anti-inflammatory effects in them (90). In particular, capsaicin dose-dependently decreased the expression of CD83 and CCR7, endocytosis of *Escherichia coli*, and the production of IL-6 and IL-12 by moDCs stimulated with pro-inflammatory cytokines. The divergent response between species might arise from dosage differences. The dose of capsaicin was 150 µM in the mouse study, while it was only 1 µM in the study performed with human DCs. A subsequent study showed that TRPV1 functions as a calcium channel and induces the release of calcitonin-gene related peptide (CGRP) upon capsaicin exposure in mouse splenic DCs (91). CGRP attenuated LPS-stimulated CD11c^+^ splenic DC responses, as it was shown by their reduced expression of CD80/CD86, decreased production of TNF and increased release of IL-10. Capsaicin also significantly reduced IFN-γ secretion in whole spleen cell cultures under Th1 polarizing conditions. These data suggest that capsaicin might contribute to immune regulation via CGRP-mediated suppression of DC activation (91).

Recent preclinical studies indicate that capsaicin treatment is able to ameliorate autoimmune disease symptoms. In an experimental model of autoimmune neuropathy, orally administered capsaicin reduced sciatic nerve demyelination and inflammatory cell infiltration when given prophylactically (92). Similarly, 0.075% capsaicin skin cream alleviated DSS-induced colitis symptoms including colon shortening, diarrhea and weight loss in mice while improved their epithelial barrier integrity and gut microbiota composition (93).

It has been known for many years that capsaicin binds to TRPV1, specifically within a ligand-binding pocket formed by transmembrane segments (94). Experimental evidence also indicates that capsaicin might interact with the ATP binding site of molecular chaperone heat-shock protein 90 (Hsp90), a key protein that stabilizes and activates signaling molecules involved in inflammation, such as NF-κB and MAPK pathways. By inhibiting Hsp90, capsaicin may disrupt these pro-inflammatory signaling cascades, thereby reducing inflammatory responses (95). Molecular docking studies suggest that capsaicin may also interact with other signaling proteins, including Abelson tyrosine-protein kinase (c-Abl), c-Src kinase, p38 MAP kinase, and VEGF receptor, that all play key roles in propagating inflammatory signals (96). *In silico* studies further predict that capsaicin can bind to and potentially modulate the activity of key inflammatory mediators such as COX-2, IL-6, and TGF-β (97), supporting its immunomodulatory properties. These interactions ranging from well-established TRPV1 binding to computationally predicted protein targets underpin the complex role of capsaicin in immune regulation and inflammation.

### 3.8 Berberine

Berberine is an isoquinoline alkaloid found in some plants like goldenseal, goldthread and Oregon grape. Its most abundant natural source is barberry (*Berberis* species), traditionally used in Asian medicine to treat fever, infections, digestive disorders, and other pathologies (98). Both *in vitro* and *in vivo* studies show that berberine possesses strong anti-inflammatory and immunomodulatory activities, and thus propose the use of berberine as a therapeutic agent for the treatment of inflammatory disorders (99).

In LPS-treated human moDCs, berberine reduced CD40, CD80 and CD86 expression, and lowered IL-1β, IL-6 and TNF production, impairing their capacity to prime Th17 responses (100) In LPS-stimulated mouse BM-DCs, berberine inhibited the secretion of TNF and IL-12, as well as the production of IL-6 and TGF-β that supposedly contributed to the inhibition of Th1 and Th17 polarization, respectively. The authors further suggest that berberine exerts its effect through inhibiting dopamine receptor-mediated signaling pathways (100). In DC2.4 cells, berberine greatly increased the secretion of IDO and TGF-β that might contribute to the ability of DCs to induce Treg differentiation, while decreasing Th17 proliferation (101). Additional data indicate that berberine is able to inhibit Th17 responses both directly and indirectly through repressing DC functions (102).

Berberine-mediated suppression of Th1 and Th17 responses has been further substantiated in animal models of type I diabetes (103) and MS (104). In addition, berberine was also shown to ameliorate DSS-induced colitis symptoms as well as Th1 and Th17 responses in mice (100). In the context of DSS-induced murine ulcerative colitis (UC), berberine inhibited colon damage and restored mucosal barrier homeostasis by inhibiting the infiltration and activation of immune cells (105). Berberine treatment decreased the percentage of inflammatory cells including DCs, Th1 and Th17 cells in the mesenteric lymph nodes and lamina propria of DSS-treated mice. In addition, berberine reduced serum levels of inflammatory cytokines including TNF, IL-1β, IL-6 and IFN-γ, and increased enteric glial cell functions (105). In streptozotocin-induced diabetic retinopathy (DR) berberine lowered serum levels of glucose, TNF, IL-1β, IL-6 and IL-17 (101). In the spleen and lymph nodes of DR mice, berberine reduced the frequency and activation of DCs. In addition, berberine lowered the ratio of Th17/Tregs indicating that berberine can suppress DC activation and influence T cell differentiation as well. These results are in line with a previous report (102), showing that berberine might affect T cell responses both directly and indirectly through DCs.

Recent *in silico* studies have indicated IKKα, as a primary molecular target of berberine (106). Additionally, berberine demonstrated strong binding affinity to the allosteric sites of AMP-activated protein kinase (AMPK) α- and β-subunits, which could contribute to the modulation of inflammatory responses as well (107). Together, these molecular interactions highlight the potential of berberine to suppress pro-inflammatory signaling and regulate DC functions.

### 3.9 Ginsenoside

Ginsenosides are a class of steroid glycosides, and triterpene saponins found exclusively in the roots of Panax species such as ginseng (*Panax ginseng*), traditional used in East Asian to enhance physical and mental performances (108). In the last few decades, the major bioactive compounds of ginseng have been shown to modulate various immune cells including DCs (109).

More than 200 structurally diverse ginsenosides exist, and most of them are categorized in the groups of protopanaxadiol (e.g. Rb1, Rc, Rd, Re, Rg3, Rg5) and protopanaxatriol (e.g. Rg1, F4, Rg6) types of glycosides (110). An early study evaluated 21 different ginsenosides for their ability to modulate LPS-triggered IL-12 production of BM-DCs, finding ginsenosides Rg6 and F4 the most effective (111). Another study demonstrated that ginsenoside Rg1 more effectively reduced LPS-triggered IL-6 and TNF production by murine DC2.4 cells than ginsenoside Rb1 (112). Interestingly, the inhibitory effects of ginsenosides Rg1 and Rb1 were diminished when the two compounds were combined (112). A fraction of ginsenosides containing predominantly Rc, Rg3, Rd and Rb1, decreased CD40, CD80, CD86, and MHC II expression by human LPS-stimulated moDCs (113). These ginsenosides also suppressed the ability of *Staphylococcus aureus*-primed moDCs to induce naïve CD4^+^ T cell proliferation and IFN-γ production. Ginsenoside metabolite compound K (CK), the main deglycosylated metabolite of ginsenosides, decreased CD80, CD86 and MHC II expression on mouse BM-DCs as well as their capacity to prime T cell proliferation *in vitro* (114). Ginsenoside Rg5 could greatly increase the efferocytotic capacity, the clearance of apoptotic cells by BM-DCs from *db/db* mice, via inhibiting Solute Carrier Family 7 Member 11 (SLC7A11), a negative regulator of efferocytosis (115). The authors further found that ginsenoside Rg5 inhibited SLC7A11 activity via direct binding.

*In vivo*, ginsenoside Rg5 promoted wound healing in the skin of diabetic (*db/db*) mice by increasing efferocytosis by DCs (115). Furthermore, CK decreased the proportion of DCs in the lymph nodes of collagen induced arthritis mice by lowering CCL21 levels in the lymph nodes and CCR7 expression on the surface of DCs (114). However, various ginsenosides can alleviate disease symptoms in mouse (116) and rat models (117) of MS; their impact on DCs has remained unexplored.

Molecular docking analyses demonstrated that ginsenoside Rb1 can bind to TLR4 (118), while ginsenoside Rf was identified as a potential ligand for peroxisome proliferator-activated receptor gamma (PPARγ), a nuclear receptor that plays an important role in regulating inflammation by controlling the expression of COX-2 (119). Additionally, molecular docking and thermal shift assays confirmed the interaction between CK and Annexin A2. This interaction prevented Annexin A2 from binding to the NF-κB p50 subunit and their nuclear co-localization, thereby attenuating NF-κB activation and downstream gene transcription (120).

## 4 Discussion

Since time immemorial, plants have been extensively used to alleviate pain and treat different type of illnesses around the world (121). Traditionally, different parts of plants including roots, leaves, seeds and fruits have been used in herbal remedy preparation. In the last few decades, it has been revealed that the bioactive compounds found in plants, also called phytochemicals, are responsible for their pharmacological actions. Research has also shown that a multitude of plant-derived bioactive compounds have significant promise in preventing and curing chronic illnesses. In addition, many phytochemicals have been recognized for their immunomodulatory activity and significant contribution to the maintenance of the body’s homeostasis (122).

Despite the fact that phytochemicals are widely distributed in fruits, vegetables and herbs, diet is insufficient to reach therapeutic levels, mainly due to their fast metabolism in the gut and liver. Moreover, phytochemicals have limited bioavailability due to their poor solubility and stability. Besides, the dietary intake form may also affect the bioavailability of phytochemicals (123). Therefore, new delivery systems and formulation techniques are under development to increase the stability, the bioavailability, and thus the therapeutic efficacy of plant-derived bioactive compounds (124). Nevertheless, the application of plant-derived compounds for therapeutic purposes is still in its infancy and faces many different challenges. In particular, *in vitro* studies show that mostly large doses (in the µM range) are required to elicit the beneficial effects of some phytochemicals that is hard to achieve *in vivo*. Moreover, high concentrations of certain phytochemicals might already have adverse and cytotoxic effects (125). For instance, in murine models, relatively high concentrations of capsaicin promoted DC activation (89), in contrast to the anti-inflammatory effects in human moDCs at markedly lower doses (90). These divergent outcomes likely arise from species-specific differences in DC subset sensitivity and the dose-dependent immune-modulatory properties of capsaicin. Moreover, berberine was shown to induce apoptosis in murine BM-DCs and splenic DCs in a dose-dependent manner (from 2 to 50 µM) while not affecting other immune cell types such as macrophages, B and T cells (126). Actually, this feature of berberine could be exploited for therapeutic purposes. Intraperitoneal injection of berberine to CIA mice alleviated disease symptoms, most probably due to its ability to selectively induce apoptosis of DCs, and thus significantly reduce their proportion in the spleen and lymph nodes of CIA mice (126). These studies underscore the current lack of consensus on the optimal dose of phytochemicals for therapeutic application; therefore, further studies are needed to assess their clinically relevant levels.

The administration route is another factor that needs to be optimized to ensure the effectiveness and safety of plant bioactive compounds. In general, orally delivered phytochemicals are poorly absorbed, since many plant-derived compounds are conjugated with glucuronide, sulfate or glutathione moieties in the gut epithelium or liver, and then excreted in urine and bile (123). Therefore, intraperitoneal administration is preferred over the oral route to avoid the potential degradation of biological agents in *in vivo* animal experiments (127). In relation to plant-derived compounds, a study showed that intraperitoneal injection of berberin led to a better anti-arthritic effect than its oral administration (126). Nevertheless, intraperitoneal injection is rarely used in the clinics, thus the efficacy of other forms of administration routes such as intravenous or subcutaneous needs to be explored for plant-derived compounds. It is more likely, though, that improving the delivery efficiency into the target tissue by nanoformulation might be the key to overcome these limitations (128). For instance, a recent study shows that gingerol encapsulation within lipid nanoparticles enhanced its stability and thus promoted its osteogenic, chemopreventive, and antibacterial properties in 3D-printed bone scaffolds (129). Another study demonstrated that β-cyclodextrin inclusion technology could enhance the stability and bioavailability of 6-shogaol (130) and curcumin (131) as well. Furthermore, encapsulation of resveratrol in nanostructured lipid carriers not only increased its stability and maintained its activity, but also decreased the necessary dose for inhibiting DC functions (52). The potency of nanocarries in DC-based immunotherapy has been extensively reviewed elsewhere (132, 133).

Another promising strategy to improve the bioavailability and thereby enhance the efficacy of phytochemicals is their combined use. Piperine acts as a broad-spectrum bioenhancer by increasing the intestinal absorption of several phytochemicals, which consequently amplifies their anti-inflammatory effects. For instance, co-administration with piperine has been shown to augment the anti-inflammatory potential of quercetin (73). Specifically, the combined administration of quercetin and piperine in the form of reconstituted oil bodies significantly improved the suppression of inflammatory cytokine production by DCs, even at low doses. Piperin is also documented to increase the bioavailability of curcumin (134). It must also be noted that enhancing the intestinal absorption of phytochemicals such as curcumin is particularly important from the perspective of DCs, as they are abundant in the gut mucosa and actively sample antigens from the intestinal lumen. Consequently, increased local bioavailability of phytochemicals in the gut allows them to directly modulate the function and phenotype of intestinal DCs, potentially shaping mucosal immune responses more effectively. Further studies in murine RAW264.7 cells suggest that ginsenoside Rg1 can synergize with glucocorticoids to enhance their anti-inflammatory effect (135). Additionally, immunomodulatory synergy has been reported between curcumin and capsaicin (97), curcumin and resveratrol (136), as well as EGCG and quercetin (137). Furthermore, curcumin showed synergistic anti-cancer effects when combined with apigenin (138). However, to date, no specific *in vitro* studies have directly evaluated the effects of these combinations on DCs.

Another challenge associated with phytochemicals is that plant-derived compounds might affect several cell types at the same time when administrated systemically. The phytochemicals introduced in this review exert significant inhibitory effects on DC functions; however, several studies showed that various phytochemicals are able to directly affect other immune cell types such as T cells and macrophages. These data suggest that plant-derived compounds probably target ubiquitous cellular signaling pathways that might lead to unexpected or undesired pharmacological effects. Although, *in vivo* animal studies showed that several phytochemicals might be effective in the treatment of autoimmune diseases associated with aberrant DC activation, the precise molecular mechanism underlying their activity needs to be explored.

*In silico* analyses have already predicted multiple molecular targets for these phytochemicals through approaches such as molecular docking and direct binding simulations. Notably, molecular targets include several components of the NF-κB signaling cascade, as well as key pro-inflammatory enzymes such as LOX and COX, whose activity can be attenuated through direct ligand binding. In addition, several of these phytochemicals play a crucial role in activating the antioxidant system through direct binding to NRF2, SOD and inhibiting the Keap1. Such interactions offer a plausible mechanistic basis for the anti-inflammatory and anti-oxidant phenotypes documented *in vitro* and *in vivo*. Furthermore, these observations underscore the complex interplay between phytochemicals and multiple signaling pathways, highlighting the necessity of further studies to precisely define their molecular targets and mechanisms of action. Understanding these multifaceted interactions will be crucial for harnessing phytochemicals as targeted therapeutics for immune-mediated diseases while minimizing their off-target effects.

**Figure 1:**
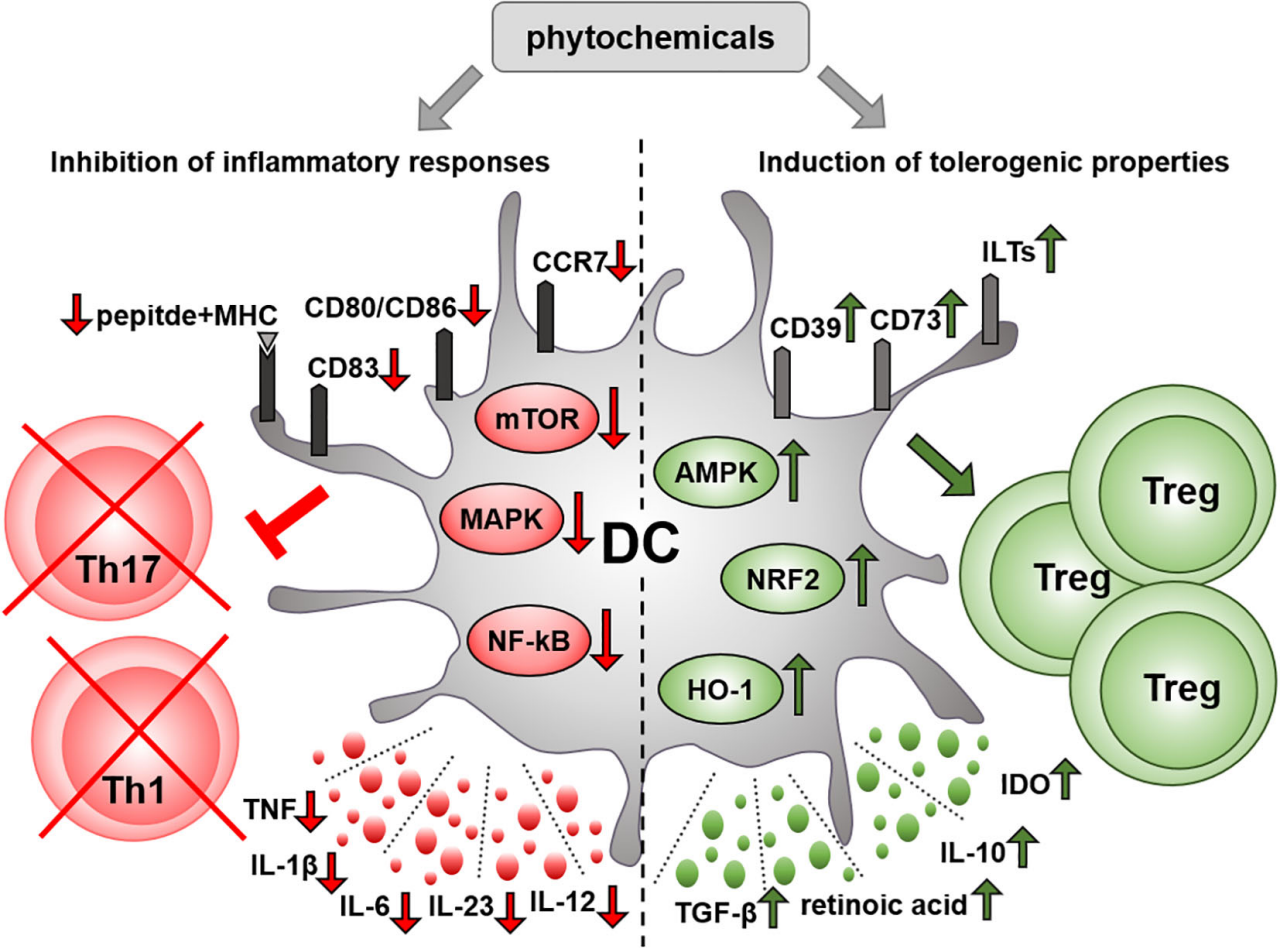
Immunomodulatory effect of phytochemicals on DCs. Various phytochemicals can inhibit the immunostimulatory function of DCs through multiple mechanisms. On the one hand, phytochemicals may impair the ability of DCs to respond to inflammatory stimuli. In particular, they can suppress the production of inflammatory mediators, and downregulate the expression of costimulatory and antigen-presenting molecules by interfering with key inflammatory signaling pathways in DCs. On the other hand, plant-derived bioactive compounds may promote a tolerogenic phenotype in DCs, characterized by upregulated expression of tolerogenic cell surface markers and increased production of anti-inflammatory mediators. Several phytochemicals suppress the capacity of DCs to induce inflammatory T helper cell subsets, such as Th1 and Th17, while increasing their ability to drive the differentiation of regulatory T cells. AMPK, AMP-activated protein kinase; CD, cluster of differentiation; DC, dendritic cell; HO-1, heme oxygenase 1; IDO, indoleamine 2,3-dioxygenase, IL, interleukin; ILT, immunoglobulin-like transcript; MAPK, mitogen activated protein kinase; MHC, major histocompatibility complex; mTOR, mammalian target of rapamycin; NF-κB, nuclear factor-kappa B; NRF2, nuclear factor-erythroid 2-related factor 2; TGF, transforming growth factor; Th, T helper cell; TNF, tumor necrosis factor; Treg, regulatory T cell.

Accumulating evidence indicates that multiple plant-derived compounds can modulate DC activity, including cytokine secretion and their T cell-priming capacity (**Figure 1**). Therefore, phytochemicals could be utilized to the generation of tolerogenic DCs for DC-based therapies that holds significant potential for the treatment of autoimmune diseases. Although, numerous protocols have been developed to manipulate the functionality of DCs prior to re-introduction into patients, to date, clinical outcomes have not been satisfactory. There are still many challenges in DC therapy that needs to be resolved such as the delivery root, optimal cell number and type of DC to be used. Additionally, a prerequisite for tolerogenic DCs is to maintain their suppressive phenotype in inflammatory environments. Therefore, the protocols for generating tolerogenic DCs also include an activation step using LPS or a cocktail of inflammatory cytokines. This also contributes to the upregulation of CCR7, which is necessary to the migration of DCs to the lymph nodes and thus to the modulation of T cell responses. Nevertheless, tolerogenic DCs generated with the immunosuppressive agents such as vitamin D3, dexamethasone or rapamycin have reduced CCR7 levels and thus impaired capacity to migrate to lymph nodes (16). Although, migration is a significant attribute of DC functionality, relatively low number of studies examined the effects of plant-derived compounds on DC migration. Given the variability and abundance of biologically active compounds in plants, it is also conceivable, that some phytochemicals are able to induce tolerogenic characteristics in DC without inhibiting their migratory potential. It is also important to mention that similar to most DC clinical trials, studies examining the effect of phytochemicals on human DCs have been carried out with moDCs, which have an inherent low migratory ability and thus rather orchestrate local immune responses (9, 16, 139). In comparison to moDCs, cDCs have a superior capacity to migrate to lymph nodes and present antigens to naive T cells (140). Thus, in future experimental studies it would be desirable to investigate the effects of plant-derived compounds on circulating DC types, especially on conventional DCs, which might serve as a more potent alternative to moDCs for DC vaccination (140).

**Figure 2:**
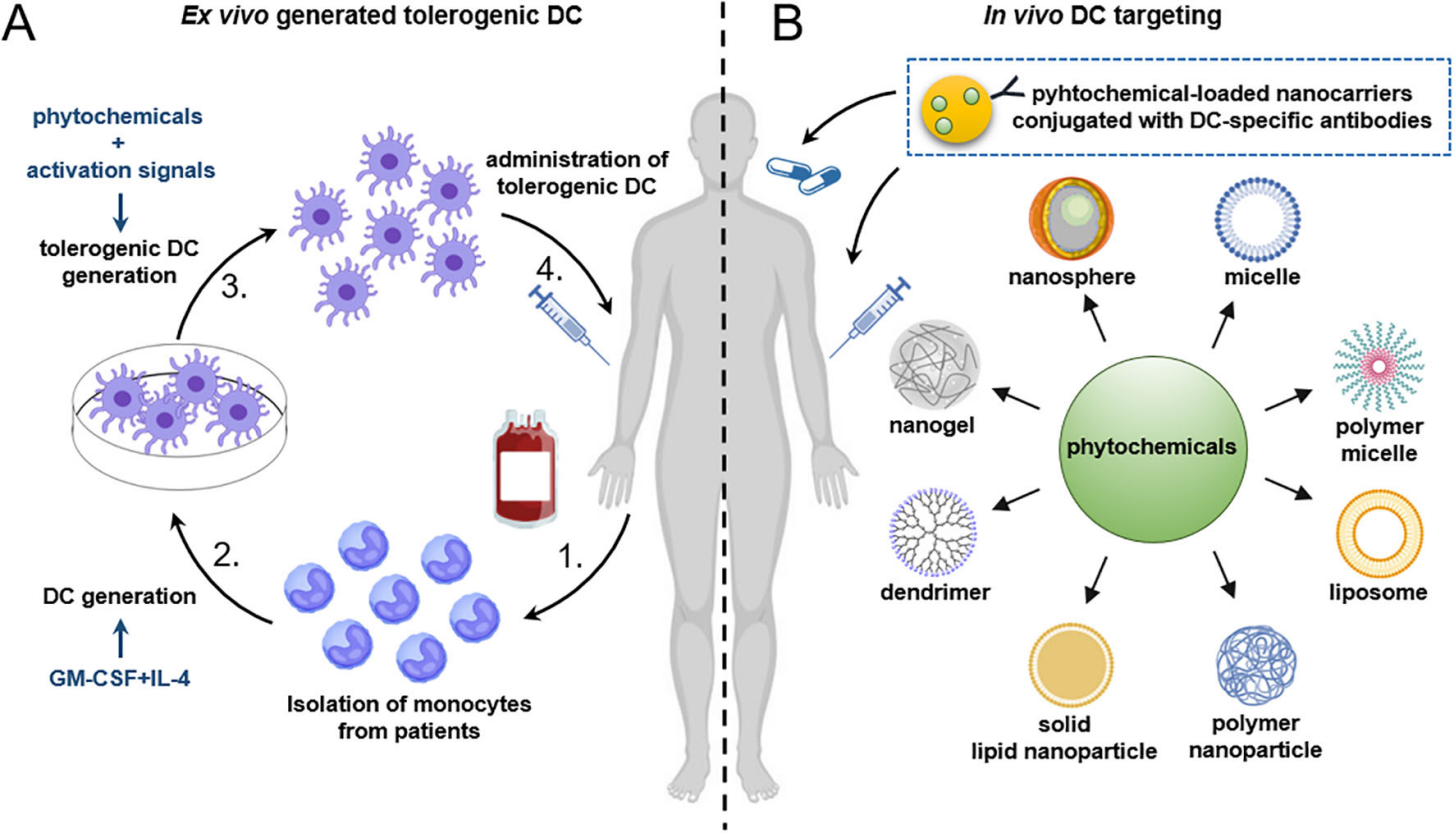
Future strategies for the generation of tolerogenic DCs using plant-derived compounds. **(A)** For the *ex vivo* generation of tolerogenic DCs, CD14^+^ monocytes are isolated from peripheral blood of patients with autoimmune diseases and then differentiated into DCs in the presence of GM-CSF and IL-4. A tolerogenic state in DCs can be achieved by the addition of plant-derived bioactive compounds. To ensure the stability of tolerogenic DCs under inflammatory conditions, activation stimuli may also be applied. Alternatively, DCs can be pulsed with disease-relevant antigens as well. Finally, the tolerogenic DCs are reintroduced into the patient. **(B)** A promising future strategy involves the *in vivo* targeting of DCs using nanoparticles loaded with phytochemicals alone, or in combination with autoantigen-associated peptides. Alternatively, nanoparticles might be loaded with antibodies against specific surface antigens expressed on DCs to enhance their targeting specificity. Finally, nanoparticles loaded with immunomodulatory agents such as phytochemicals can be administered intravenously, subcutaneously, or via non-invasive routes such as oral delivery. DC, dendritic cell; GM-CSF, granulocyte-macrophage colony-stimulating factor; IL-4, interleukin-4.

In conclusion, plant-derived bioactive compounds might open up new avenues in the treatment of autoimmune diseases. Several clinical trials of phytochemicals including curcumin, EGCG, resveratrol, apigenin and berberine for the treatment of various autoimmune diseases have been recently completed or are still ongoing that indicates the potential clinical benefits of these compounds (**Table 3**.). Alternatively, several plant-derived bioactive compounds might serve as potential tools for the generation of DC-based vaccines (**Figure 2**). In addition to the phytochemicals introduced in this review, many others might have the ability to modulate DC responses that could be exploited for therapeutic purposes. For instance, a recent study demonstrated that even a rose flavor compound, namely β-damascone, was also able to suppress DC-mediated immune responses, and thus to ameliorate contact hypersensitivity in mice (141). All these data imply that the plant kingdom is one of the richest sources of bioactive compounds with pharmaceutical activity, and holds a great potential for the discovery of new therapeutic agents.

**Table 3:** Phytochemicals for autoimmune diseases in clinical trials.

Phytochemical	Disease	Dose/Administration route	Phase	Status	References
curcumin	MS	500 mg curcumin orally twice a day for 24 months	2	Completed	NCT01514370 (143)
curcumin (Bioglan)	SLE	a tablet containing curcumin (632 mg) - piperine (15,800 mg) once a day for 3 months	2	Completed	NCT05430087
curcumin (Norflo Oro)	Autoimmune Uveitis	2 single foil pouches Norflo Oro (curcumin-phospholipid 600 mg) orally per day for 12 months	1	Completed	NCT03584724 (144)
curcumin (Longvida™)	RA	4 capsules twice a day (4 g per day) for 4 months	1	Completed	NCT00752154
curcumin	lupus nephritis	1000 mg Curcumin Oral Capsule	2	Completed	NCT05714670
curcumin	Hashimoto’s thyroiditis	500 mg curcumin capsules 3 times per day for 3 months	Not Applicable	Active	NCT05975866
EGCG (Sunphenon®)	MS	200–800 mg (1–4 capsules) orally for 30 months	2, 3	Completed	NCT00799890 (145, 146)
EGCG (Polyphenon E)	MS	2 capsules of Polyphenon E (200 mg EGCG) twice a day for 12 months	2	Terminated*	NCT01451723 (147)
EGCG	MS	600 mg of EGCG and 60 ml of coconut oil per day for 4 months	2	Completed	NCT03740295
EGCG (Sunphenon®)	MS	200 mg of EGCG twice daily, after 3 months 400 mg twice daily for 18 months	1, 2	Completed	NCT00525668
resveratrol	type I diabetes	1500 mg trans-resveratrol	Not Applicable	Completed	NCT03436992
resveratrol	type I diabetes	500 mg of oral trans-resveratrol twice daily for 12 weeks	1	Active	NCT04449198
resveratrol	RA	1 tablet (1000 mg) once a day for 3 months	Not Applicable	Active	NCT07089381
apigenin	RA	1 capsule containing 10 mg apigenin) and 1 capsule containing 50 mg glycyrrhizin twice daily for 6 months	Not Applicable	Active	NCT05788705
berberine	Latent autoimmune diabetes in adults	0.6 g (6 pills) of Berberine tablets and 0.6 g (6 pills) of Inulin tablets twice a day orally for 3 months	4	Active	NCT04698330

Trade name is given in parentheses where available.

EGCG, Epigallocatechin-3-gallate; MS, multiple sclerosis; RA, Rheumatoid arthritis; SLE, systemic lupus erythematosus.

*Terminated due to unusual high frequency of elevated liver function tests.
